# The association of weather and mortality in Bangladesh from 1983–2009

**DOI:** 10.3402/gha.v5i0.19121

**Published:** 2012-11-23

**Authors:** Nurul Alam, Wietze Lindeboom, Dilruba Begum, Peter Kim Streatfield

**Affiliations:** 1 Centre for Population, Urbanization and Climate Change, icddr,b, Dhaka, Bangladesh; 2 Cardialysis, Rotterdam, The Netherlands

**Keywords:** weather, temperature, rainfall, mortality, rural, Abhoynagar, Bangladesh

## Abstract

**Introduction:**

The association of weather and mortality have not been widely studied in subtropical monsoon regions, particularly in Bangladesh. This study aims to assess the association of weather and mortality (measured with temperature and rainfall), adjusting for time trend and seasonal patterns in Abhoynagar, Bangladesh.

**Material and methods:**

A sample vital registration system (SVRS) was set up in 1982 to facilitate operational research in family planning and maternal and child health. SVRS provided data on death counts and population from 1983–2009. The Bangladesh Meteorological Department provided data on daily temperature and rainfall for the same period. Time series Poisson regression with cubic spline functions was used, allowing for over-dispersion, including lagged weather parameters, and adjusting for time trends and seasonal patterns. Analysis was carried out using R statistical software.

**Results:**

Both weekly mean temperature and rainfall showed strong seasonal patterns. After adjusting for seasonal pattern and time trend, weekly mean temperatures (lag 0) below the 25th percentile and between the 25th and 75th percentiles were associated with increased mortality risk, particularly in females and adults aged 20–59 years by 2.3–2.4% for every 1°C decrease. Temperature above the 75th percentile did not increase the risk. Every 1 mm increase in rainfall up to 14 mm of weekly average rainfall over lag 0–4 weeks was associated with decreased mortality risks. Rainfall above 14 mm was associated with increased mortality risk.

**Conclusion:**

The relationships between temperature, rainfall and mortality reveal the importance of understanding the current factors contributing to adaptation and acclimatization, and how these can be enhanced to reduce negative impacts from weather.

The effect of weather on health is undeniable. Interest in the effect of weather change on health is increasing, especially in the light of global concern for potential climate change. Climate change affects every aspect of the society, from the health of the global economy to the health of our children (Ban Ki-Moon, UN Secretary General, speech 24 May 2009). It echoes the importance of research on mitigation and adaptation to climate change in vulnerable and resource-limited countries. Bangladesh has been found to be one of the most vulnerable countries to the adverse effects of climate change (1). Planning interventions require evidence. Such evidence is far less than what is required for formulating mitigation, adaptation, and poverty alleviation strategies.

Weather data from the Bangladesh Meteorological Department (BMD) on temperature (minimum and maximum), rainfall, and cyclones observed in 1950–2010 exhibited a sign of climate change (2). Daily temperatures showed an increasing trend and the increase was faster for minimum temperature. Frequency of heavy rainfall in a short span of time in pre-monsoon and monsoon has exhibited a considerable increase in recent years. With ongoing climate change, extreme weather events, such as floods, drought, or cyclones, are predicted to increase in frequency and duration (3). During 1960–1990, there were two intense cyclones with wind speeds more than 200 km/hour and 3–10 m high waves in the North Bay of Bengal; however, there were seven cyclones during 1991–2010 (2). Devastating cyclones caused extensive damage to life, property, and livestock. Human sufferings in terms of food, water, shelter, health, and overall livelihood were enormous because of the low flat terrain, high population density, poorly built houses, high level of poverty, natural-resource-dependent economy, and low adaptive capacity (4). Other impacts of climate change in this low-lying delta includes inundation of arable land, salinity intrusion, reduced fresh water, and persistence of transboundary pests and diseases (4–6). Furthermore, drinking water from natural sources in coastal areas has become contaminated by varying degrees of salinity due to salt water intrusion from rising sea levels, cyclone and storm surges, and upstream withdrawal of freshwater and affected health indicated by excess hypertension in pregnancy (6).

The association between high and low temperature and mortality has been investigated in several studies. The heterogeneity of the effects of temperature across geographic, climatic, and cultural zones was evident. In a multi-country ecological comparison in Europe, higher excess mortality rates were found in less severe, milder winters, where, all else being equal, there should be less potential for cold strains and cold-related mortality (7, 8). In 16 European cities characterized by different weather conditions, analysis reveals that in both summer and winter the strongest effects were observed in the Mediterranean cities, where winter is less severe or milder (9). These studies reveal that, in developed countries, the lower the temperature range the stronger the temperature–mortality relationship at low temperature. The effect of temperature variation in subtropical monsoon regions is unknown.

Weather in Bangladesh with its subtropical climate varies considerably between regions. On the basis of entire climatic conditions, the country is divided into seven distinct climatic zones (17). The International Centre for Diarrhoeal Disease Research, Bangladesh (icddr,b), has set up longitudinal vital registration systems in Matlab and Abhoynagar in 1966 and 1982, respectively, for conducting operational research in family planning, maternal, and child health. These two rural areas are in two climatic zones, which provide a rare opportunity to examine the weather–mortality relationships in different climatic conditions. Matlab is located in the south-central zone characterized by more frequent and severe hail storms, nor'westers, and tornadoes, and Abhoynagar is located in the south-western zone characterized by higher dew-rate. According to the BMD data, during 1983–2009 temperature and rainfall differ substantially; Abhoynagar showed both lower minimum temperatures (5.0°C vs. 8.6°C) and higher maximum temperatures (43.2°C vs. 37.8°C) than Matlab. Average rainfall in Abhoynagar was 4.8 mm with standard deviation 14.3, lower than 5.8 mm with a standard deviation of 16.4 in Matlab. The highest rainfall in a single day was 255 mm in Abhoynagar, lower than 334 mm in Matlab.

With respect to temperature and rainfall, Abhoynagar is different from Matlab, but no study has so far examined the weather–mortality relationships other than in Matlab. One study showed seasonal patterns of deaths in Matlab and another study reported that daily mortality increased with low temperatures in the preceding weeks and no association between high temperature and daily mortality during the period 1994–2002 (10, 11). These studies were limited to the association between temperature and mortality, excluding rainfall. The present study will examine the temperature– and rainfall–mortality relationships in Abhoynagar where temperature varies more and rainfall less than those in Matlab.

## Objectives

The overall objectives of this study are to investigate the effects of temperature and rainfall on all-cause mortality in different age and sex groups in Abhoynagar and to compare the results with those of studies carried out in other climatic zone in Bangladesh (12).

## Methods and data

This study used total deaths and total population at risk in Abhoynagar subdistrict and weather data from a nearby weather station of the BMD in Jessore district. Abhoynagar is predominantly rural, located in Khulna division in the southwest of Bangladesh, between Jessore and Khulna cities – about 30 km away from each (13). Khulna division reached the replacement level of fertility around the year 2000. A sample vital registration system (SVRS) was set up in the division by icddr,b in late 1982 to conduct operational research in the areas of family planning, infant, child, adolescent and maternal health, and health equity. SVRS covered 122 villages in 7 out of 17 unions selected randomly since late 1982 and another 32 villages in 2 unions since early 1984. A household listing operation was carried out in selected villages to prepare the sampling frame. The systematic random sampling was used to select every sixth household in sampled villages to prepare a sociodemographic profile of the households for surveillance. Trained field research assistants visited sampled households in 3-monthly rounds to record vital events; births, deaths, migrations, and marriages and marital disruptions. Two field research supervisors supervised their data collection activities on a regular basis. The vital events recorded were edited for consistencies and added to the longitudinal relational database.

BMD is responsible for observation, recording, and archiving of climate data for various stations in the country. BMD continuously uses weather data for monitoring time trends. We retrieved daily maximum and minimum temperature and rainfall from Jessore weather station for 1983–2009. Daily temperature or rainfall, if missing, was replaced by the estimate derived from the linear interpolation. Mean temperature was calculated as the average of minimum and maximum temperature of the day.

### Statistical analysis

During the period 1983–2009 (or 9,862 days), the Abhoynagar SVRS recorded 4,850 deaths. Weeks rather than days was chosen as unit of analysis to minimize fluctuations due to small number. The relationship between the average weekly temperature (minimum, maximum, and mean) and average weekly rainfall with weekly death count was examined using graphics followed by generalized additive Poisson regression models with cubic spline functions, allowing for overdispersion. The model is expressed with the formula:

$$\begin{array}{l} {\text{Mortality}}_{\text{t}}{\text{ – Poisson(mean}}_{\text{t}}\text{)}\\ {\text{mean}}_{\text{t}}{\text{= b}}_{\text{0}}{\text{ + s(time}}_{\text{t}}{\text{; df = 4 per year) + s(temperature}}_{\text{t}}{\text{; df = 10) + s(rainfall}}_{\text{t}}\text{; df = 10)} \end{array}$$

where t denotes time, df denotes degrees of freedom, and s denotes a cubic spline function. Models were fitted to the average weekly death count, to weekly mean temperature of up to 3 weeks (lag 0–%3B3) and rainfall up to 4 weeks prior to the week of death (lag 0–4), to assess the effects of low or high temperature or low or high rainfall over longer periods. Combined time trend and seasonal pattern were included with four unpenalized degrees of freedom for seasonal patterns and trends per year. The exposure response to meteorological factors was penalized, allowing a maximum of 10 degrees of freedom.

## Results

During the observation period of 27 years (or 1,409 weeks), the population of the Abhoynagar surveillance site increased from 21,547 during the first week of 1983 to 34,774 during the last week of 2009. During this period, SVRS registered an average of 3.4 deaths per week. Infants accounted for 26% of all deaths and the elderly (aged 60 years and above) for 44%, totaling 70%. During this period, the lowest weekly minimum temperature observed was 14.3°C and the highest weekly maximum temperature was 34.3°C. Average daily rainfall was 4.8 mm with a peak of 255 mm in a single day.

**Fig. 1:**
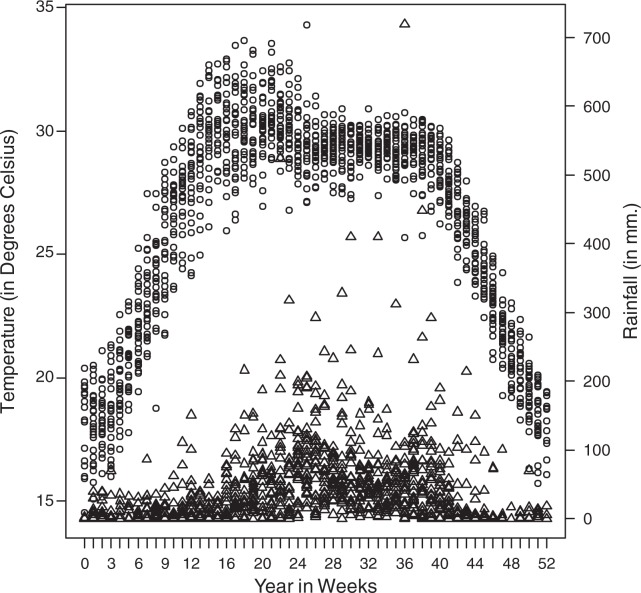
Annual pattern of weekly temperature (circle) and rainfall (pyramid), 1983–2009

Weekly temperature showed a seasonal pattern, with the peak in April–May and the lowest at the beginning and the end of the calendar year (Fig. 1). Overall, temperature and rainfall are positively correlated (*r*=0.09, *p*<0.01); however, during the rainy season (June–September) rainfall has a moderating effect on temperature (*r*=−0.49, *p*<0.001).

**Fig. 2:**
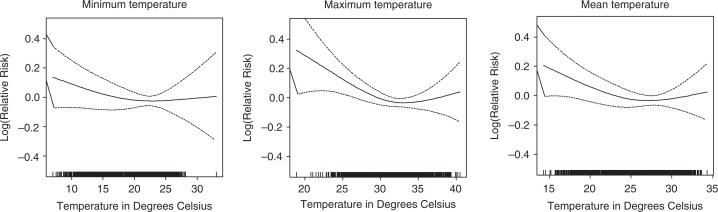
Association of temperature and mortality, after adjusting for trend and seasonality.

The temperature–mortality associations are displayed in Fig. 2 for average weekly minimum, maximum, and mean temperature. Maximum temperature showed the strongest graphical association with mortality followed by mean temperature. Generalized linear Poisson regression models show that weekly mean temperature had the strongest association with weekly mortality; below 23.0°C, the relative mortality risk increased by 2.3% with every 1°C decrease in temperature, and between 23.0 and 29.6°C, the relative risk increased by 2.4% with a 1°C decrease (Table 1). Above 29.6°C, higher temperature was also associated with a lower mortality risk (relative risk=−2.3%).

**Table 1:** Linear approximation of the association of mortality with minimum, maximum and mean temperature, after adjusting for time trend and seasonality

	<25% (first quartile)			25%–75% (2nd and 3rd quartile)		>75% (last quartile)				
			
Temperature in °C	Tem^1^ <	RR^2^ (%)	95% CI^3^	RR^2^ (%)	95% CI	Tem >	RR^2^ (%)	95% CI	% DE^4^	GCV^5^
Maximum	29.6	−1.8	(−4.1, −0.5)	−2.2	(−4.1, −3.0)	34.0	0.1	(−0.2, +0.4)	13.70	1.1838
Minimum	16.1	−2.2*	(−4.1, −0.3)	−2.2***	(−3.2, −1.1)	25.7	0.3	(−0.1, +0.6)	13.30	1.1892
Mean	23.0	−2.3*	(−4.4, −0.1)	−2.4**	(−3.9, −0.9)	29.6	−2.3*	(−2.6, −2.0)	13.60	1.1858

1 Temperature.

2 Change in relative risk in percent.

3 95% confidence interval.

4 Deviance explained.

5 Geometric coefficient of variation.

* *P*<0.05

** *P*<0.01

*** *P*< 0.001

**Fig. 3:**
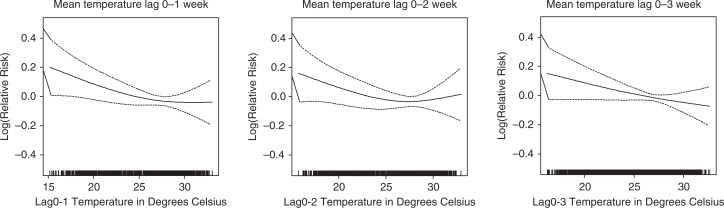
Association of mortality with mean temperature at different time lags, after adjusting for trend and seasonality.

Weekly mean temperature was included in the models to assess the temperature–mortality associations over different time lags. The average temperature over a 2-week period (temperature in the week of occurrence of death and during the preceding week) has the strongest association with weekly mortality (Fig. 3). Lag 0–1 temperature was used to assess the temperature–mortality associations by sex and age groups.

Linear approximations of the associations of lag 0–1 mean temperature with mortality were statistically significant across temperatures; below 25th percentile and between 25th and 75th percentiles (Table 2). Disaggregation of the temperature–mortality association by sex and age reveals sex and age differences in the temperature effect. Low temperature (below 75th percentile) was associated with increased mortality risk of females, but not males. The low-temperature–mortality risk was significantly higher for age groups 5–19 and 20–59 years. This was not the case for infants, children 1–4 years, and elderly (60+ years). Temperature above 75th percentile was not associated with mortality risk of any sex and age group. Mortality of females increased by 4.3% with every 1°C decrease in temperature below the 25th percentile and increased by 3.8% with every 1°C decrease in temperature between the 25th and 75th percentile. For the age group 5–19 years, the temperature–mortality association below the 75th percentile was opposite the direction of the temperature–mortality association for the age group 20–59 years. A detailed study would be needed to understand the reverse temperature–mortality relationship.

**Table 2:** Associations of lag 0–1 mean temperature and mortality for sex and age groups, after adjusting for trend and seasonality, 1983–2009

	Tem^1^<25% (first quartile)		Tem^1^ 25%–75% (2nd and 3rd quartile)		Tem^1^>75% (last quartile)			
			
Sex and age group	RR^2^ (%)	95% CI^3^	RR^2^ (%)	95% CI	RR^2^ (%)	95% CI	% DE^4^	GCV^5^
Overall	−2.5*	(−3.5, −1.5)	−2.4**	(−3.9, −0.8)	−0.1	(−0.4, +0.2)	13.60	1.1858
Male	−0.5	(−3.6,+2.6)	−1.0	(−3.2, +1.2)	−0.3	(−0.7, +0.2)	6.02	1.1757
Female	−4.3**	(−7.3, −1.3)	−3.8**	(−5.9, −1.5)	0.0	(−0.5, +0.4)	9.27	1.2252
Age group								
Infant	−3.5	(−10.7, +4.2)	−0.9	(−6.2, +4.7)	0.1	(−0.8, +1.1)	10.40	0.6085
1–4	−3.5	(−10.7, +4.2)	−0.9	(−6.2, +.7)	0.1	(−0.8, +1.1)	10.40	0.6085
5–19	9.0*	(0.7, +18.1)	7.6*	(1.6, +13.9)	−0.2	(−1.1, +0.7)	13.60	1.1858
20–59	−6.3**	(−10.6, −1.7)	−4.8**	(−8.0, −1.5)	0.2	(−0.4, +0.9)	3.61	1.1054
60+	−1.0	(−4.3, +2.3)	−2.0	(−4.3, +0.4)	−0.3	(−0.8, +0.2)	4.85	1.2109

1 Temperature.

2 Change in relative risk in percent.

3 95% confidence interval.

4 Deviance explained.

5 Geometric coefficient of variation.

* *P*<0.05

** *P*<0.01

*** *P*<0.001

**Fig. 4:**
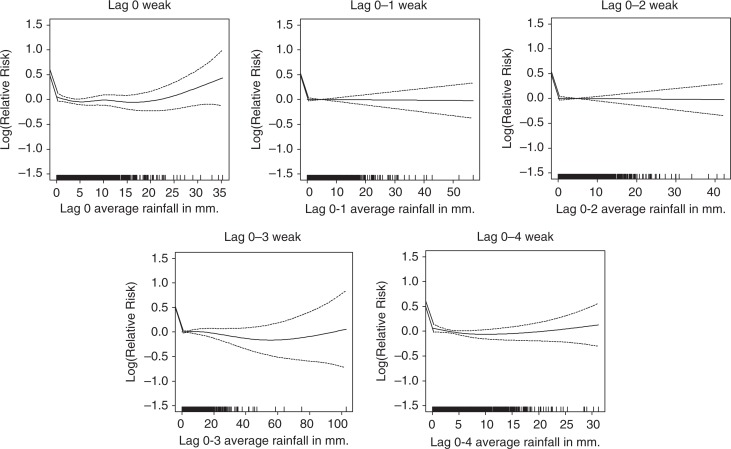
Association of mortality with rainfall at different time lags, after adjusting for trend and seasonality.

Weekly mean rainfall was included in the models to assess the rainfall–mortality associations over different time lags (Fig. 4). Mortality as smoothed functions of rainfall, using 3 mm (59th percentile) and 14 mm (91st percentile) as cut-off values for these rainfall models, showed significant associations for the longer time lags. The optimal rainfall model seemed to be the lag 0–4 model (up to 4 weeks preceding the week of death) with, below 3 mm rainfall, a relative mortality risk of −7.0% (95% CI: −11.7%, −2.1%); between 3 and 14 mm rainfall, a relative risk of −1.7% (95% CI: −2.7%, −0.7%); and above 14 mm rainfall a relative mortality risk of 1.2% (95% CI: 0.1%, −2.2%) (Table 3).

**Table 3:** Associations of mortality with lag 0–4 rainfall for sex and age groups, after adjusting for trend and seasonality, 1983–2009

	Rainfall<3 mm		Rainfall 3–14 mm		Rainfall>14 mm			
			
Sub group	RR^1^ (%)	95% CI^2^	RR^1^ (%)	95% CI	RR^1^ (%)	95% CI	% DE^3^	GCV^4^
Overall	−7.0***	(−11.7, −2.1)	−1.7***	(−2.7, −0.7)	1.2*	(0.1, +2.2)	17.20	1.1275
Male	−1.8	(−8.2, +5.1)	−0.9	(−2.1, +0.2)	1.2•	(−0.2, +2.5)	5.04	1.1925
Female	−14.0***	(−19.6, −7.9)	−2.8***	(−3.9, −1.6)	1.4•	(−0.1, +2.9)	8.19	1.2288
Age group								
Infant	−4.3	(−13.4, +5.7)	−1.4	(−3.1, +0.4)	1.0	(−1.0, +3.0)	16.0	1.2438
1–4	3.8	(−11.8, +22.1)	2.2	(−0.5, +5.0)	3.8*	(0.7, +6.9)	13.0	0.61279
5–19	15.7•	(−0.4, +34.3)	4.0**	(1.3, +6.8)	−2.3	(−5.2, +0.6)	8.65	0.68735
20–59	−10.5*	(−19.6, −0.3)	−2.2*	(−4.0, −0.4)	2.0•	(−0.1, +4.1)	1.75	1.1068
60+	−12.0**	(−18.5, +5.0)	−3.0***	(−4.2, −1.7)	1.7*	(0.2, +3.3)	4.14	1.2463

1 Change in relative risk in percent.

2 95% confidence interval.

3 Deviance explained.

4 Geometric coefficient of variation.

• *P*<0.1

* *P*<0.05

** *P*<0.01

*** *P*<0.001

Gender disaggregated models showed a reduction in female mortality risks below 14 mm of average rainfall with every 1 mm increase in rainfall and increased mortality risks of both males and females (*p*<0.05) at rainfall levels over 14 mm (Fig. 4 and Table 3). The rainfall–mortality associations also depend on age of the individuals. The association for the age group 5–19 years was opposite the associations for the age groups 20–59 and 60+ years. Mortality risks of the adults (aged 20–59 years) and elderly decreased with every 1 mm increase in rainfall below 14 mm rainfall and increased above 14 mm rainfall.

## Discussion

This study showed, after adjustment for time trend and seasonality, a moderate but significant increase in all-cause mortality at lower temperature in Abhoynagar compared to a more marked increase in Matlab (11, 12). Compared to Matlab, temperature (minimum and maximum) in Abhoynagar was more extreme. This suggests that variations in temperature influence the temperature–mortality relationship at lower temperatures. This finding is consistent with the findings of several studies. In Europe, countries with the mildest winter climates exhibited the highest excess winter mortality than countries with severe winter climates (7–9). The heterogeneity of the effect reflects the capacity to adapt to extreme temperature. Data on cross-country thermal-efficiency standards in housing indicated that those countries with poorest housing demonstrated the highest excess winter mortality and poorest housing was more common in countries with mildest winter climate (7). The thermal efficiency of the house is perhaps on the causal pathways between low temperature and mortality risk.

The temperature–mortality relationships between these two areas in different climatic zones may be due to difference in weather and adaptation than other health-related factors. Communities naturally adapt – physiologically, culturally, and behaviorally – to living in cold and warmer climates. Common adaptive measures that match with large temperature variation at microlevel are thermal efficiency in housing and clothing. Available data on the quality of housing revealed that more houses in Abhoynagar have walls and roofs with cement/concrete than in Matlab. In 2008 in Abhoynagar, 30% of the houses were roofed and 41% were walled with cement/concrete compared to 2% houses roofed and 4% walled with cement/concrete in Matlab in 2005 (13, 14). In winter, the indoor temperature at night in houses roofed and walled with tin or straw is the same as the outdoor temperature. Cold temperature accompanied by chill and fog makes people sick as many do not have enough cold-protective measures. However, data on possession of winter clothes at individual and household levels are not available in either area. The finding emphasizes the need to revisit local adaptive measures to explain and combat cold-related mortality in the community.

There was a gender difference in temperature–mortality relationship in Abhoynagar. Why females were more vulnerable at lower temperature needs more in-depth investigation. In rural areas, women are usually homemakers and men are the main income earners (13, 14). This gender-based division of labor might have played a role in increasing vulnerability of females. Women do washing and cleaning for all household members. Such frequent and prolonged washing and cleaning with cold water in cold weather may have contributed to the increased mortality risk in females at lower temperature. Gender difference in treatment seeking cannot be ruled out.

Abhoynagar with less rainfall and less variation (daily average 4.8 mm ranging from 0 to 255 mm) exhibited the rainfall–mortality relationship, which was not the case in Matlab with more rainfall and large variation (daily average 5.8 mm ranging from 0 to 334 mm). In Abhoynagar, every 1 mm increase in rainfall up to 14 mm of weekly average rainfall over lag 0–4 weeks decreased the mortality risk and above 14 mm increased the risk. This finding is consistent with the finding that in Dhaka, every 10 mm increase in rainfall above the threshold of 45 mm of average rainfall over lags 0–8 weeks was associated with increased weekly number of hospital visits due to cholera and non-cholera diarrhea in 1996–2002 (Hasizume 2007, 2008). Many infectious diseases, for example, diarrhea and outbreaks of Giardia and Cryptosporidium, reach their peak during the rainy season (15, 16). Rainfall may have contributed to pathogenic contamination of drinking water, causing water-borne disease, and such contamination may differ between Matlab and Abhoynagar.

These two areas, Matlab and Abhoynagar, are comparable in terms of demography of mortality statistics. For example, under-five mortality rates in Matlab comparison area and Abhoynagar were 46 and 42, respectively, in 2009 (10, 12). The life expectancy at birth in Matlab in 2009 was 68.9 years for males and 71.5 years for females, and in Abhoynagar, it was 70.4 years for males and 71.6 years for females in 2008–2009. However, the fertility rate was a little higher in Matlab area than in Abhoynagar (TFR=2.5 vs. 2.2). As expected, younger population (age below 15 years) was higher by 5% in Matlab than Abhoynagar. The difference in the weather–mortality relationship between the two areas at 142 km (linear distance) apart may not be influenced very much by the difference in demographic factors.

Such as no effect of rainfall on mortality in Matlab compared to the positive effect of moderate rainfall and the negative effect of high rainfall on mortality in Abhoynagar. Some of the differences may be attributed to the small sample in Abhoynagar. Weekly death counts regressed on weekly average temperature and rainfall may have smoothed out some short-term effects. Another limitation is that the distance from the study area to the nearest weather station is not close by and is 30 km away in Jessore. Despite these various limitations, the spatial differences in the effects of temperature and rainfall on mortality reveal the importance of studying local adaptation and the acclimatization process for a better understanding of the community responses to weather variation and the weather_mortality relationship. Further studies on cause-specific mortality may reveal details about the origin of susceptibilities and differences.
